# Synchronous Gastric Metastasis From Sigmoid Colon Cancer: A Case Report

**DOI:** 10.1002/ccr3.72513

**Published:** 2026-04-12

**Authors:** Seung Jong Oh, Seul‐Gi Oh, Sun Keun Choi

**Affiliations:** ^1^ Department of Surgery, Inha University Hospital Inha University College of Medicine Incheon South Korea; ^2^ Department of Surgery, Asan Medical Center University of Ulsan College of Medicine Seoul South Korea

**Keywords:** case report, neoplasm metastasis, sigmoid neoplasm, stomach neoplasm, synchronous

## Abstract

Colorectal cancer is the third most common cancer worldwide, and as such is a significant global health concern. Distant metastases of colorectal cancer to the lung, liver, and bone are well documented, while gastrointestinal tract (GIT) metastases are very rare. Herein, we report a case of gastric metastasis from sigmoid colon cancer. A 78‐year‐old male was diagnosed with both gastric and colorectal cancer, which occurred synchronously. The presence of two primary cancers was diagnosed, and radical subtotal gastrectomy and anterior resection were performed simultaneously. The postoperative pathology report showed a moderately differentiated adenocarcinoma in the sigmoid colon and stomach. Immunohistochemical (IHC) analysis revealed that the resected gastric specimens showed tumor cells that were positive for both cytokeratin 20 (CK20) and caudal‐type homeobox gene 2 (CDX2) and negative for CK7. This confirmed that the gastric lesion was a metastasis from colorectal cancer. Clinicians should be aware of the potential presence of metastatic gastric cancer, whether synchronous or metachronous, in other solid organ malignancies.

## Key Clinical Message

A rare case of synchronous gastric metastasis from sigmoid colon cancer is presented. Immunohistochemistry (CK20+, CDX2+, CK7‐) confirmed the gastric lesion originated from colorectal cancer, highlighting the need to consider metastatic gastric involvement in patients with other primary malignancies of solid organs.

## 1 Introduction

Colorectal cancer is the third most common cancer worldwide and the second leading cause of cancer‐related mortality in the world [1]. Approximately 20% of patients with colorectal cancer already have metastases at the time of initial diagnosis, with the liver (50%) and lung (10%–15%) being the most commonly involved sites of metastasis from colorectal cancer. Primary cancers from other organs rarely metastasize to the stomach. The rarity of stomach metastasis from colorectal cancer is further supported by a large‐scale autopsy study, which reported a range of 0.7%–5.4% for gastric metastases across various cancers, indicating that colorectal cancer contributes only minimally within this range [2]. Here, we report a notable case of synchronous metastasis to the stomach in a sigmoid colon cancer patient.

## 2 Case Presentation

Chief complaints and history of present illness: A 78‐year‐old male underwent upper and lower endoscopy as part of a routine health examination.

History of past illness: The patient had Alzheimer's disease.

Laboratory examinations: The carcinoembryonic antigen (CEA) level was 0.87 ng/mL.

**FIGURE 1:**
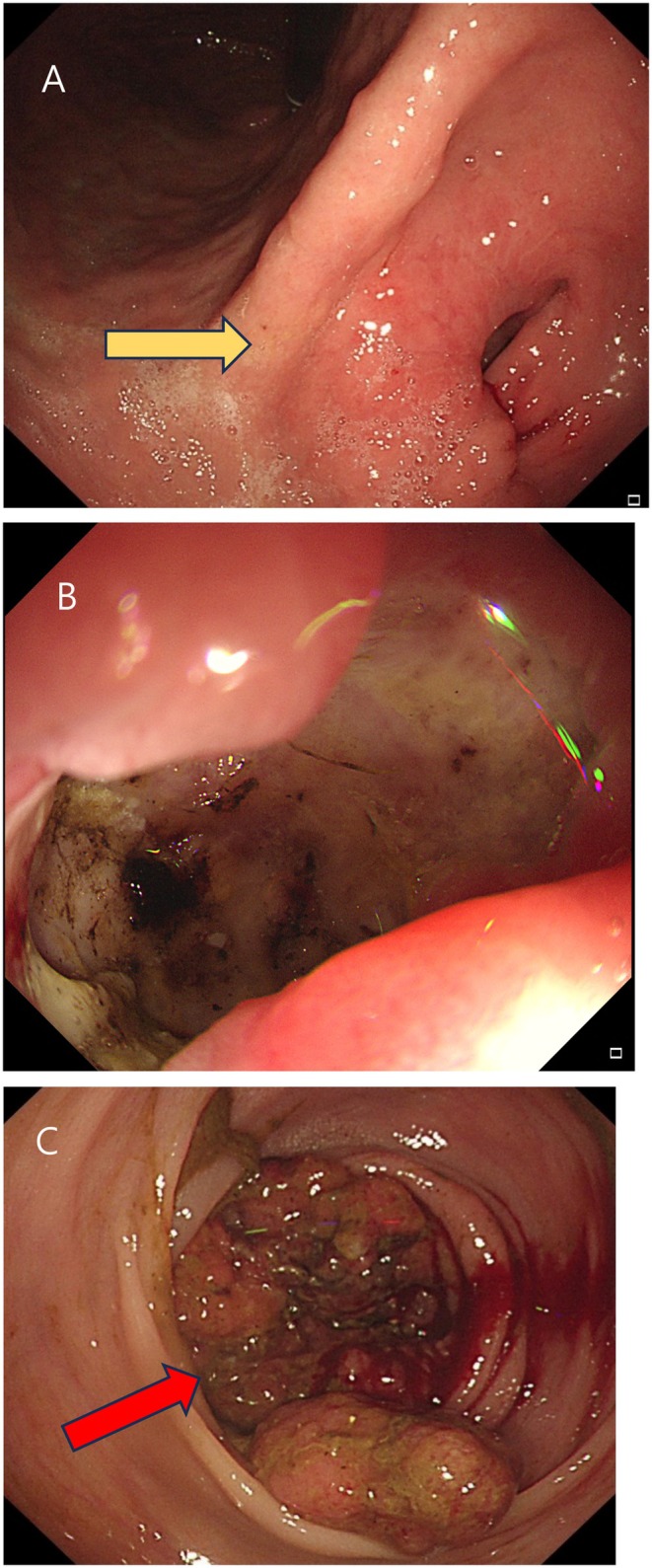
Esophagogastroduodenoscopy image showed hard, consistent mass in the lesser curvature of mid antrum (yellow arrow) (A). Upon closer inspection, an ulcerofungating mass is revealed (B). Colonoscopy image showed 12 cm from the anal verge; there is an ulcerofungating mass which encircles the entire lumen (red arrow) (C).

**FIGURE 2:**
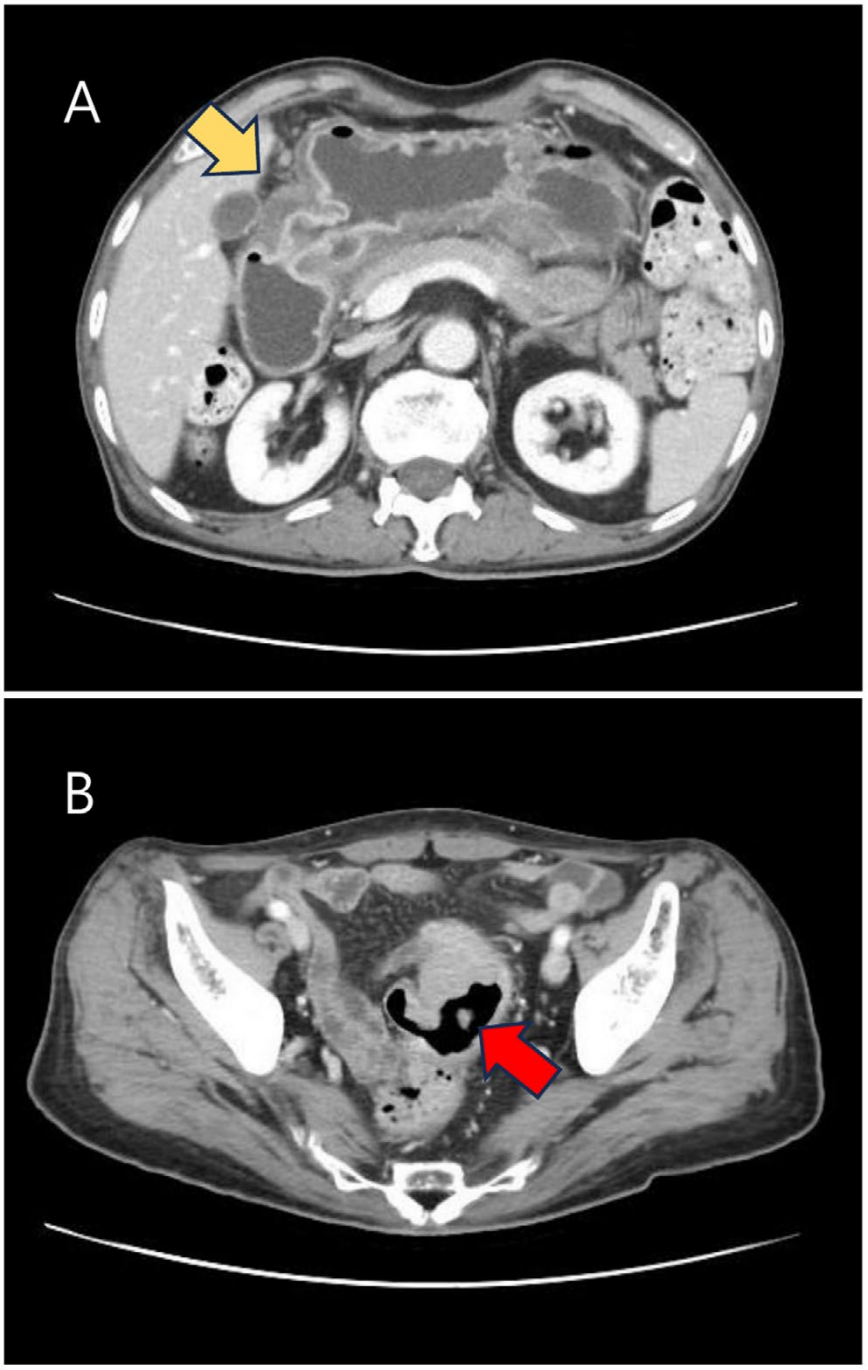
Abdominal computed tomography scan shows stomach cancer involving the antrum to pylorus with peri‐gastric infiltration (yellow arrow) (A) and distal sigmoid colon cancer with peri‐colonic infiltration (red arrow) (B).

**FIGURE 3:**
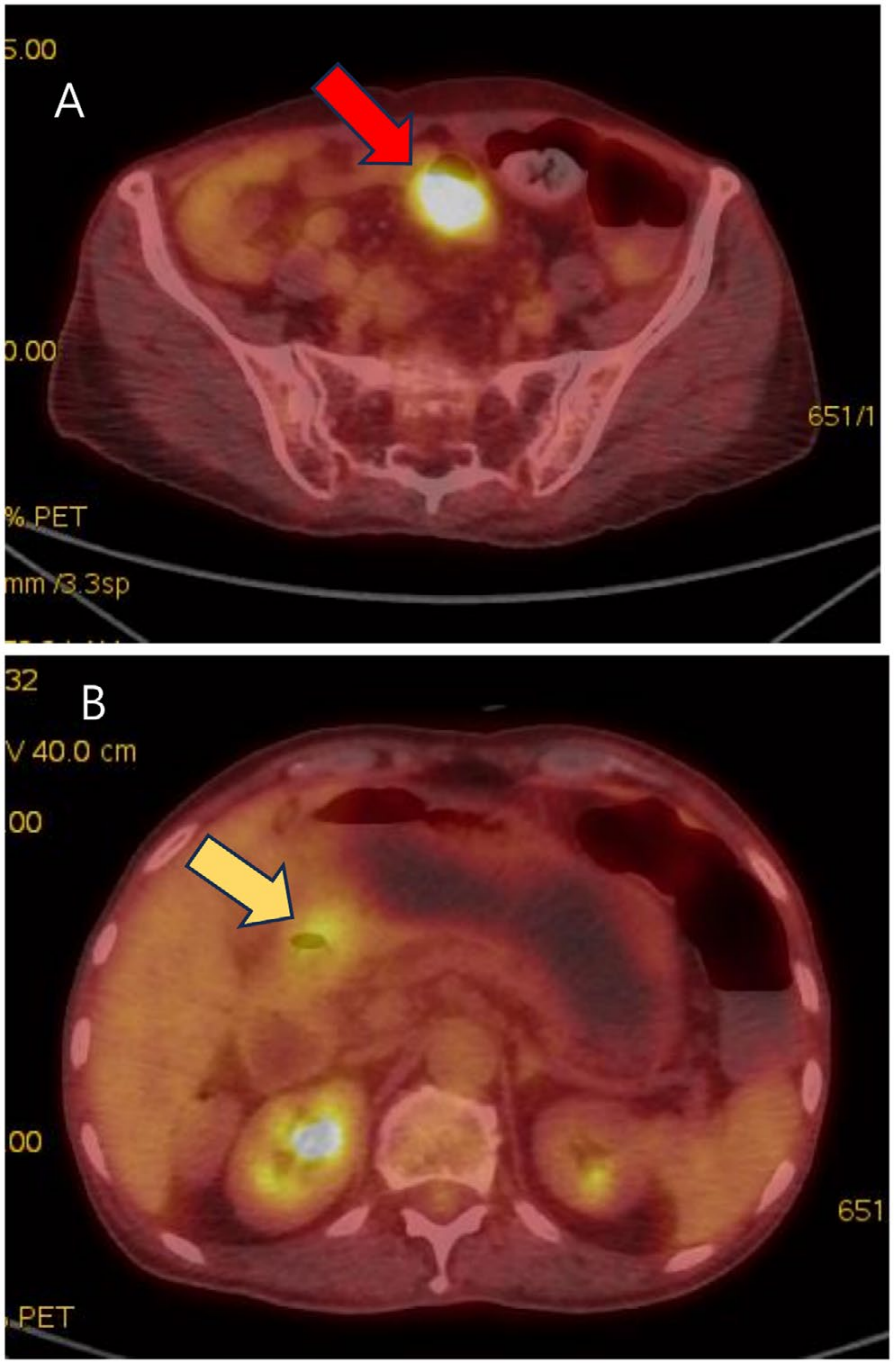
A PET/CT scan revealed significant FDG uptake localized to the primary sigmoid colon mass (red arrow) (A) and the metastatic lesion in the gastric antrum (yellow arrow) (B). No other abnormal FDG accumulation was observed in distant organs or the peritoneum.

Imaging examinations: The esophagogastroduodenoscopy (EGD) revealed a 3 × 2 cm ulcerofungating mass in the lesser curvature side of the mid antrum of the stomach (Figure 1A,B), and a colonoscopy showed a 4 × 2 cm ulcerofungating mass in the distal sigmoid colon, located 12 cm from the anal verge (Figure 1C). The pathological examination of the endoscopic biopsy specimens obtained from the stomach and sigmoid colon indicated well‐differentiated adenocarcinoma, which showed similar histologic characteristics with each other. The abdominal computed tomography (CT) scan revealed a tumor involving the antrum to pylorus with peri‐gastric infiltration (Figure 2A) and a 5.5 cm‐sized mass at the distal sigmoid colon (Figure 2B). On positron emission tomography/CT (PET/CT), abnormal fluorodeoxyglucose (FDG) uptake was exclusively localized to the primary sigmoid colon mass (Figure 3A) and the metastatic gastric lesion (Figure 3B). Notably, there was no evidence of FDG‐avid lesions in other distant organs or signs of peritoneal seeding, supporting the possibility of curative resection for both synchronous lesions.

## 3 Final Diagnosis

**FIGURE 4:**
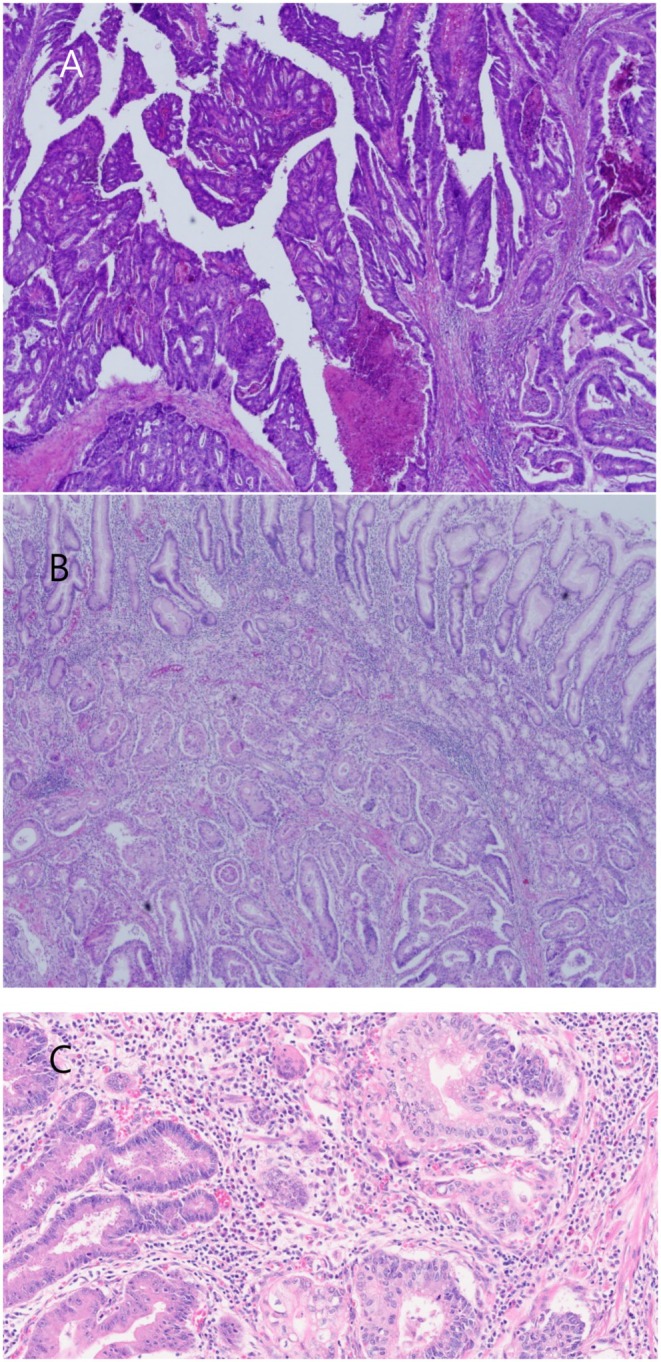
A moderately differentiated adenocarcinoma in sigmoid colon (A) and stomach (B, C).

**FIGURE 5:**
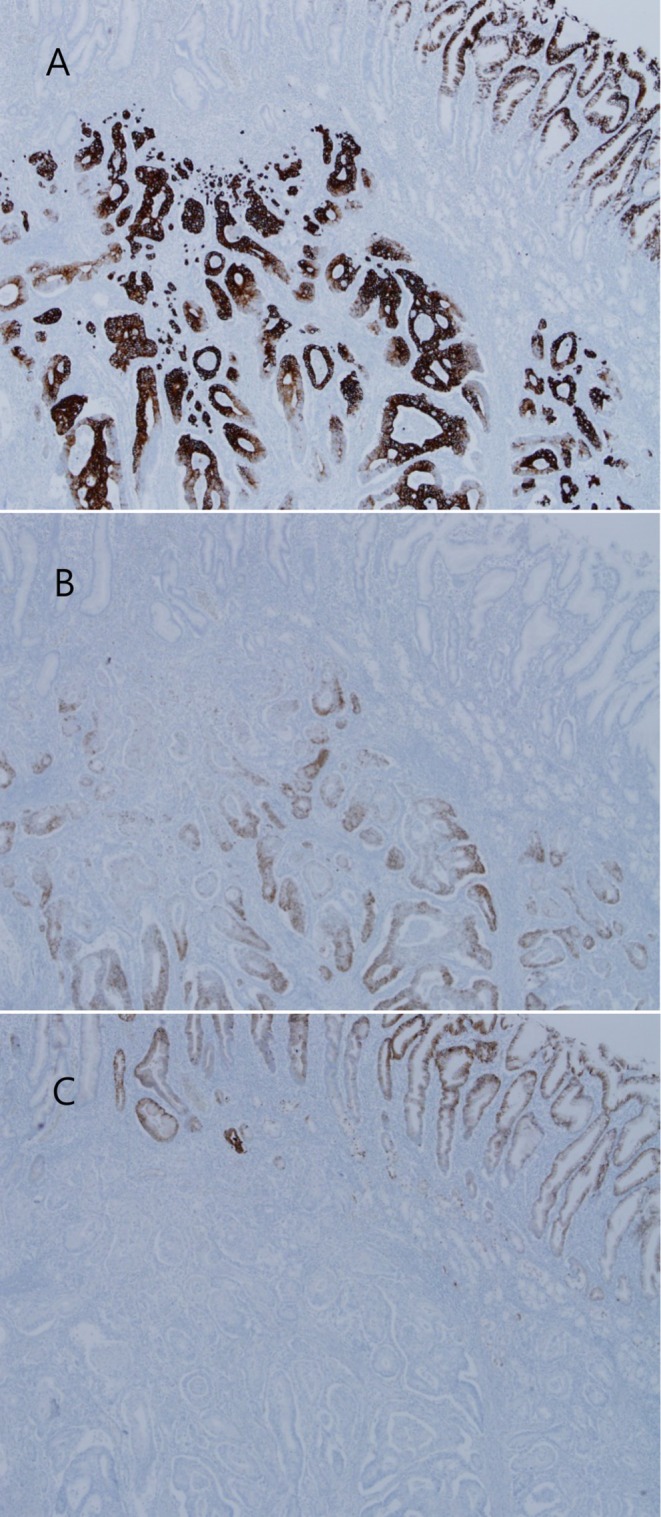
IHC analysis of gastric lesion was positive for CK20 (A) and CDX2 (B), and negative for CK7 (C).

The postoperative pathology report revealed a moderately differentiated adenocarcinoma in the sigmoid colon (Figure 4A) and stomach (Figures 4B,C). The immunohistochemical (IHC) analysis of the gastric lesion showed positive staining for CK20 (Figure 5A) and CDX2 (Figure 5B), and negative staining for CK7 (Figure 5C). These results suggest that the cancer of the sigmoid colon had metastasized to the stomach.

## 4 Treatment

The initial diagnosis of double primary cancer was made, and radical subtotal gastrectomy and anterior resection were performed simultaneously. The patient recovered uneventfully and was discharged 10 days after surgery without complications. The postoperative pathology showed gastric metastasis of sigmoid colon cancer. Postoperatively, the patient received adjuvant chemotherapy with the XELOX regimen (capecitabine and oxaliplatin). However, the treatment was discontinued after the 5th cycle due to a decline in the patient's general performance status.

## 5 Outcome and Follow‐Up

The patient has been followed up with regular imaging and endoscopy without further cytotoxic therapy for 5 years. Then this case was closed due to the absence of evidence of metastasis.

## 6 Discussion

The determination of the true incidence of metastasis to the stomach is challenging due to its low prevalence. Several studies have reported an incidence of 0.2%–0.8% [3, 4, 5, 6] and autopsy studies have reported higher incidences of 1.7%–5.4% [4, 7, 8, 9]. Studies based on autopsy results conducted on individuals with certain neoplasms are a reliable source of actual incidence data. The metastatic route from the sigmoid colon to the stomach is generally considered hematogenous, as the stomach lacks a direct lymphatic connection with the distal colon.

Gastric metastasis from colorectal cancer is rarely documented in the literature [3, 4, 8, 10] and only one case has been reported, found during a routine health check‐up with no symptoms. Patients diagnosed with gastric metastasis may present with various symptoms. Green et al. documented that among the ten cases identified through surgical biopsy, notable clinical symptoms and physical examination outcomes encompassed diffuse abdominal pain, nausea and vomiting, anorexia, guaiac‐positive stools, and gastrointestinal bleeding [4].

Previous studies have demonstrated that endoscopic patterns, including multiple nodules, bull's eye pattern, exophytic mass lesions, ulcers, and multiple tumors, are prevalent and characteristic. These findings are often accompanied by doughnut‐shaped ridged lesions and volcanic ulcers. These endoscopic patterns have been shown to be useful in the diagnosis of gastric metastases [11]. However, some cases of gastric metastasis are difficult to distinguish from primary gastric cancer [10, 12]. Hirano K et al. reported that more than half were solitary metastases. Despite the heterogeneity of the morphologies observed, approximately half of the cases exhibited a resemblance to early gastric cancer [13]. In the present study, EGD revealed an ulcerofungating mass with mucosal invasion, which was diagnosed as typical primary gastric cancer rather than metastatic gastric cancer.

In the field of cancer research, a significant body of literature has emerged on the use of immunohistochemical testing for the identification of carcinomas of unknown primary origin. A seminal study by Bayrak R et al. reported the expression of CK7 in upper GIT tumors, whereas CK20 was found to be predominantly expressed in lower GIT tumors [14]. Building on this, Park SY et al. undertook a comprehensive evaluation of multiple immunohistochemical markers for each tumor with the aim of determining the combination of the 10 markers that most effectively predicted primary sites [15]. The most predictive multi‐marker phenotypes, as determined by a combination of specificity and positive predictive value, were CDX2+/CK7‐/CK20+ for colorectal primary tumors and CDX2+/CK7+/CK20‐ for upper GI tumors. The patient in question had a CK7‐/CK20+ pattern and positive staining for CDX2. The immunohistochemical profile of CK7‐/CK20+/CDX2+ is a powerful tool for confirming colorectal origin. According to Chu et al. and Park et al., the CK7‐/CK20+ pattern exhibits a high specificity of 96.7%–98.7% for colorectal adenocarcinoma [15, 16]. While primary gastric cancer often expresses CK7 in approximately 68% of cases, it is rarely seen in colorectal cancer (7%–17%). Additionally, Bayrak et al. demonstrated that CDX2 shows a high sensitivity of 97% for colorectal primaries, further distinguishing them from other gastrointestinal malignancies [14].

Primary gastric cancer that arises in intestinal metaplasia may exhibit a colorectal‐like immunoprofile. However, existing data indicate that a CK7‐negative/CK20‐positive pattern is rare in gastric adenocarcinoma, even in cases of intestinalized mucosa [17]. Our study observed a gastric lesion against a background of intestinal metaplasia that did not express CK7. This suggests that the lesion was a colorectal adenocarcinoma that had metastasized to the stomach. Large series and panel‐based studies consistently demonstrate that most gastric adenocarcinomas retain CK7 expression with variable CK20 [18]. In contrast, the CK7‐/CK20+ phenotype is strongly enriched in colorectal adenocarcinomas and is considered characteristic of colorectal rather than gastric origin [19]. Therefore, although intestinal metaplasia can modify the gastric mucosal immunophenotype to some extent, a diffuse CK7‐negative/CK20‐positive profile in an adenocarcinoma involving the stomach is more indicative of a metastatic colorectal primary than a de novo gastric carcinoma arising in metaplastic mucosa.

In the management of metastatic colorectal cancer, curative surgery is widely accepted as the preferred method for the removal of resectable metastases in the lung or liver. However, for metastases in extremely rare sites, such as the stomach, a consensus on the optimal approach has yet to be established [20]. Nushijima et al. reported a case [21]. A 52‐year‐old woman was diagnosed with transverse colon cancer, underwent left hemicolectomy, and received adjuvant XELOX therapy for 6 months. One year and 3 months after left hemicolectomy, EGD revealed a submucosal tumor in the stomach and histology showed metastatic gastric cancer from transverse colon cancer. Radical distal gastrectomy was performed, but peritoneal dissemination and para‐aortic lymph node recurrence were noted 7 months after the second surgery. The prognosis is generally poor because gastric involvement typically indicates an advanced stage of disease and is often associated with metastases to other sites. In our case, a metastatic lesion in the stomach was diagnosed simultaneously with the diagnosis of colorectal cancer. Radical resection was performed on both sites with a favorable prognosis. Therefore, it is imperative to perform EGD in patients diagnosed with colorectal cancer.

## 7 Conclusion

Tumor metastases to distant anatomical structures can disrupt normal function and significantly increase disease morbidity and mortality. Consequently, metastases from a known primary cancer have a critical impact on staging, prognosis, and treatment strategies. In addition, cancers that initially present with distant metastases often require extensive evaluation to identify the primary site and guide optimal treatment. In conclusion, clinicians should be aware of the potential presence of metastatic gastric cancer, whether synchronous or metachronous, in other solid organ malignancies. Appropriate systemic treatment, including chemotherapy or hormonal therapy for the primary tumor, is the most desirable treatment for metastatic tumors in the stomach; however, surgical resection of metastatic gastric tumors may be recommended to improve the patient's quality of life if there is a risk of complications such as bleeding or tumor perforation.

## Author Contributions

**Seung Jong Oh:** conceptualization, data curation, formal analysis, methodology, writing – original draft. **Seul‐Gi Oh:** conceptualization, data curation, formal analysis, writing – review and editing. **Sun Keun Choi:** conceptualization, methodology, project administration, supervision, writing – review and editing.

## Funding

The authors have nothing to report.

## Ethics Statement

The authors have nothing to report.

## Consent

Study participant, or their legal guardian, provided written informed consent prior to publication of this case report.

## Conflicts of Interest

The authors declare no conflicts of interest.

## Data Availability

No datasets were generated or analyzed during the current study. All relevant information is included in this article.
